# Supplementary material for: Molecular understanding of polyelectrolyte binders that actively regulate ion transport in sulfur cathodes
Source: Nat Commun. 2017 Dec 22;8:2277. doi: 10.1038/s41467-017-02410-6 (PMC5741623; doi:10.1038/s41467-017-02410-6)
Supplement: Supplementary file 1 — Supplementary Information [file 41467_2017_2410_MOESM1_ESM.pdf]

Supplementary Methods

Free Energy Molecular Dynamics Simulations. Valence (bonds, angles, dihedral and improper torsions) and short range van der Waals interactions for the PEB, polysulfide, 1,3-dioxlane (DOL), 1,3-dioxlane (DOL) and TFSI molecules were described using the DREIDING[1] forcefield. As in our previous work[2], the electrostatic interactions were treated as Gaussian charge distributions, dynamically evaluated by means of the QTPIE fluctuating charge model[3]. Initially, we employed the original QEq parameters of Goddard and Rappe[4]. Further optimizations of the lithium and sulfur electronegativity (χ) and hardness (η) parameters were performed to reproduce the Bader charge distributions on the solvated lithium polysulfides given in our previous work[5]. Here we applied a Newton-Rapson iterative approach, minimizing the residual function (i.e., the differences in the calculated QTPIE and Bader charges for the ensemble lithium polysulfide structures). Optimal χ and η parameters for the DOL and DME molecules were obtained from equilibrium molecular dynamics simulations of the bulk liquids, matching the experimental density. Details of our optimization procedure and forcefield parameters will be published separately[6].

Various systems comprising 1 PEB-1 molecule (4 units), $TFSI^-$ and Li_2S_n were embedded in a box of pre-equilibrated 1:1 DOL:DME molecules. These systems were then subjected to molecular dynamics (MD) simulations using the LAMMPS simulation engine[7]. The system was equilibrated according to our usual approach[8–10]: briefly, after initial conjugant gradient minimization at a force tolerance of 10^{-4} kcal mol^{-1} Å^{-2}, the system was slowly heated from 0 K to 298 K with a Langevin thermostat in the constant temperature, constant volume micro-canonical (NVT) ensemble. The temperature coupling constant was 0.1 ps and the simulation time step was 1.0 fs.

This equilibration was followed by 10 ns of constant-pressure (iso-baric), constant-temperature (NPT) dynamics at the required temperature and 1 atm. The temperature coupling constant was 0.1ps while the pressure piston constant was 2.0 ps. The equations of motion used are those of Shinoda *et al.*[11], which combine the hydrostatic equations of Martyna *et al.*[12] with the strain energy proposed by Parrinello and Rahman[13]. The time integration schemes closely follow the time-reversible measure-preserving Verlet integrators derived by Tuckerman *et al.*[14]

After equilibration, the binding free energy of the polysulfide or $TFSI^{-}$ were obtained separately from well-tempered Metadynamics simulations[15,16], employing the center of mass distances as the collective variable. Here, the Metadynamics step was 0.5 ps, at which time, Gaussian functions of with 0.5 kcal mol^{-1} were added to the biasing function of the system. Metadynamics simulations of at least 100 ns were run, with the collective variable monitored to ensure ballistic behavior and that the residual functional of the free energy calculated every 10 ps converged to zero. Usually, we found convergence after 50 ps.

Simulated X-ray Absorption Spectroscopy Calculations. Systems comprising a PEB-1/LiTFSI/Li_2S_n complex was equilibrated in a box of 60 1:1 DOL:DME solvent molecules and subjected to 20 ns of equilibration NPT MD simulations followed by 20 ns of NVT MD simulations. During the last 10 ns of NVT MD simulation, snapshots of the system (atomic positions) were saved every 0.5 ns (a total of 20 snapshots for each system) and used as input into an in-house code employing constrained-occupancy DFT calculations within the XCH approximation[17–19] to calculate the XAS spectra (80 individual calculations). Plane–wave pseudopotential calculations using ultrasoft pseudopotentials[20] were performed using the PWSCF code within the Quantum-ESPRESSO package[21]. We used a kinetic energy cut-off for electronic wave functions of 25 Ry and a density cut-off of 200 Ry. The core-excited Kohn–

Sham eigenspectrum was generated using the XCH approach[18]. Based on a numerically converged self-consistent charge density, we generated the unoccupied states for our XAS calculations non-self-consistently, sufficiently sampling the first Brillouin zone at the gamma point, employing an efficient implementation of the Shirley interpolation scheme[22] generalized to handle ultrasoft pseudopotentials[23]. Matrix elements were evaluated within the PAW frozen-core approximation[24]. Core-excited ultrasoft pseudopotentials and corresponding atomic orbitals were generated with the Vanderbilt code[20]. Each computed transition was convoluted with a 0.2 eV Gaussian function to produce continuous spectra.

Due to the use of pseudopotentials in our calculations (which means that we can only reliably compare the relative calculated excitation energies), we have developed an alignment scheme based on formation energy differences between the ground and core-excited states of the system and those of an isolated atom in the same simulation cell[19,25]. Direct comparison to experiment is accomplished by first calibrating an unambiguous reference system. In the case of the sulfur compounds considered in this study, we rigidly shifted the first major peak in the sulfur K-edge XAS of an isolated S_2 molecule by +2467.5 eV to match the same in a gas phase experiment[26]. This empirical shift, is unique to the pseudopotentials employed in this study, and is applied to all subsequent calculated spectra. Previous experience has shown that this alignment scheme predicts XAS peak positions to within ~0.1 eV[17,19], which is typical of the experimental uncertainty in this energy range. It is well known that Kohn-Sham DFT within the PBE approximation underestimates band gaps[27,28] and concomitantly band-widths due to inaccurate quasiparticle (excitation) energies[29,30]. As a result, the calculated XAS spectrum is usually too narrow compared to experiments. Thus we dilated the computed XAS spectrum by 25%, as in our previous works[2,31].

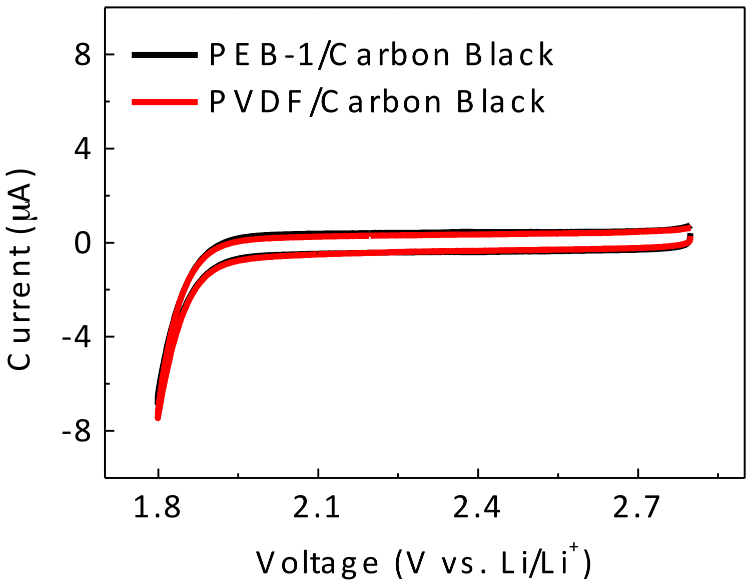

Supplementary Figure 1 | CV curves of PEB-1/carbon black and PVDF/carbon black at a scan rate of 0.1 mV s^{-1}.

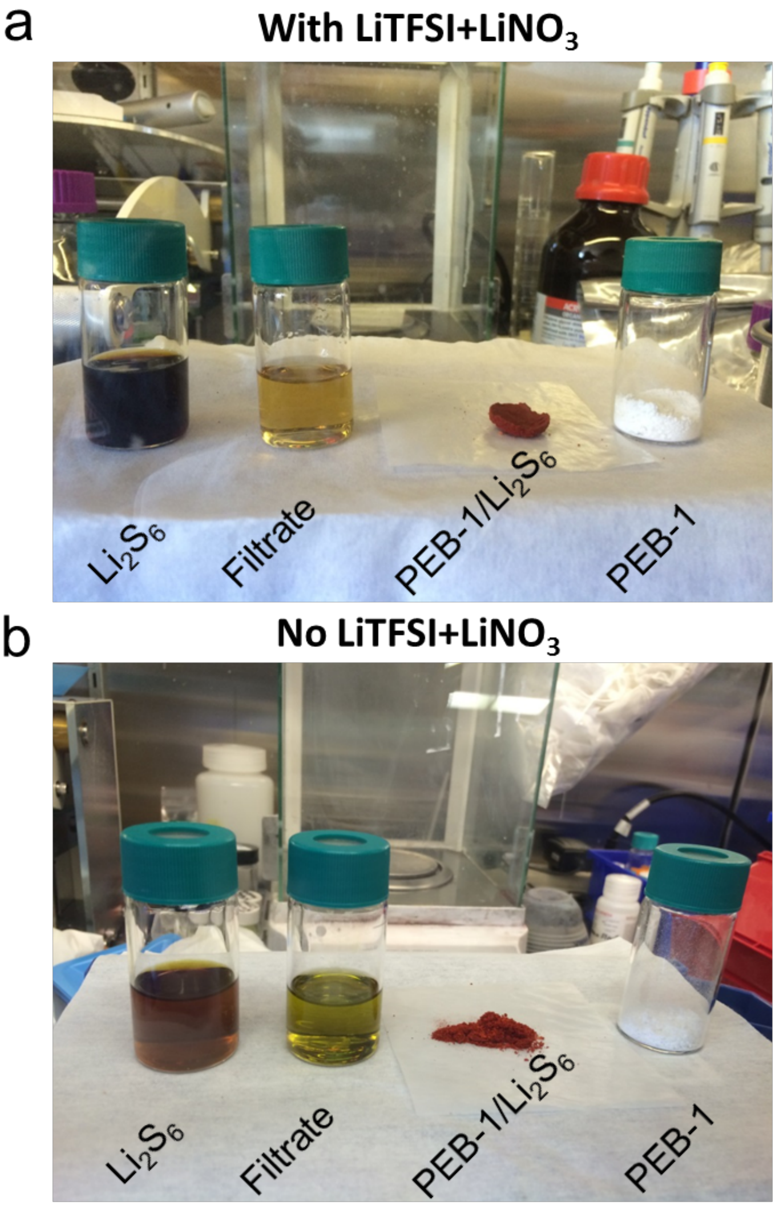

Supplementary Figure 2 | Qualitative understanding of the polysulfide adsorbing character of PEB-1. a, Electrolyte composition: 1.0 M LiTFSI + 0.2 M $LiNO_3$. **b**, Electrolyte obviates use of both LiTFSI and $LiNO_3$. From left to right: Lithium polysulfides dissolved in electrolyte (10 mM, prepared nominally as Li_2S_6), the filtrate after PEB-1 had been introduced (and isolated) to sequester polysulfides from solution, the PEB-1/S_6 composite after PEB-1 underwent anion metathesis with Li_2S_6, pristine PEB-1 powder.

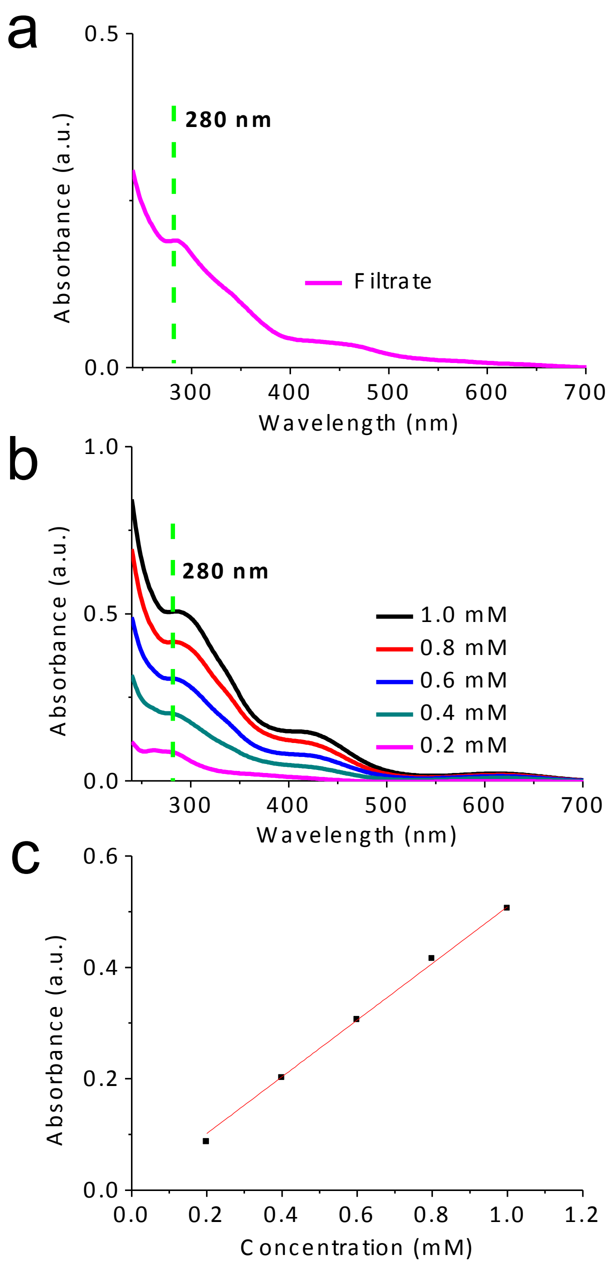

Supplementary Figure 3 | Quantitative determination of residual polysulfides in electrolyte after PEB-1 introduced as a chemisorbing species. a,UV-vis spectrum of the filtrate after anion metathesis. **b**, UV-vis spectra of standard Li_2S_6 solutions with concentrations of 0.20, 0.40, 0.60, 0.80, and 1.00 mM. **c**, Calibration curve linking optical absorbance at 280 nm to Li_2S_6 concentration.

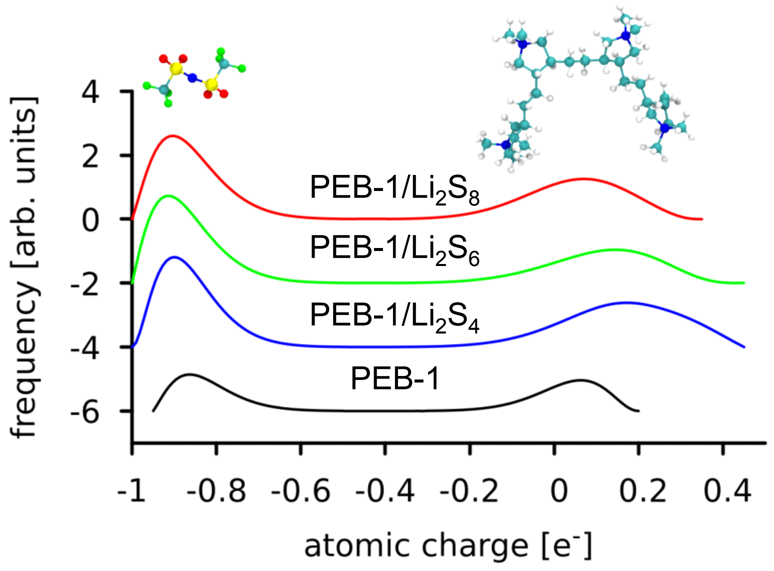

Supplementary Figure 4 | DFT calculations of the valence electron charge density of N atoms in TFSI⁻ (left hand side) and the pyrrolidinium moieties of a tetramer model of PEB-1.

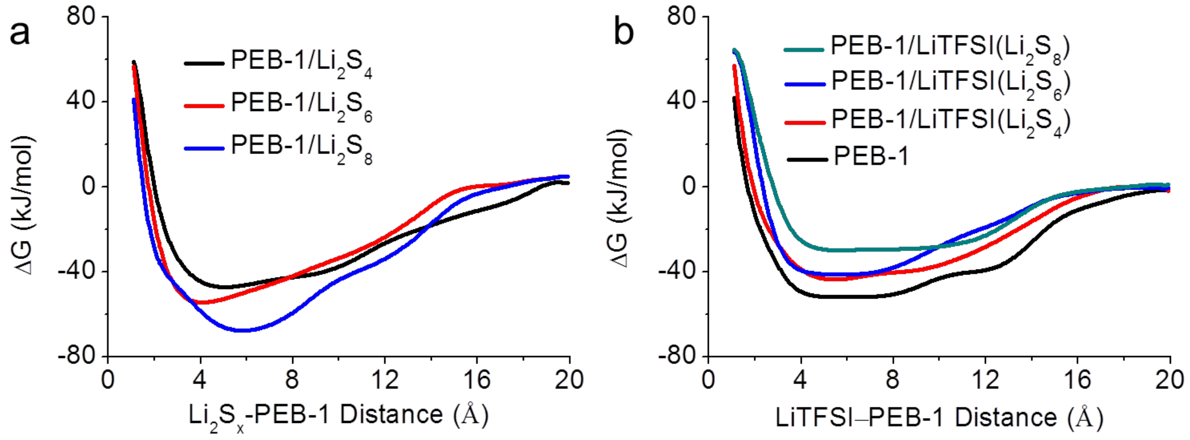

Supplementary Figure 5 | Free energy curves highlighting the selective nature of Li_2S_n and LiTFSI binding to PEB-1/S_n/LiTFSI ion clusters as it depends on oligomer length, *n*. a, The interaction of PEB-1 and Li_2S_n when LiTFSI is present. **b**, The interaction of PEB-1 and LiTFSI when Li_2S_n is present.

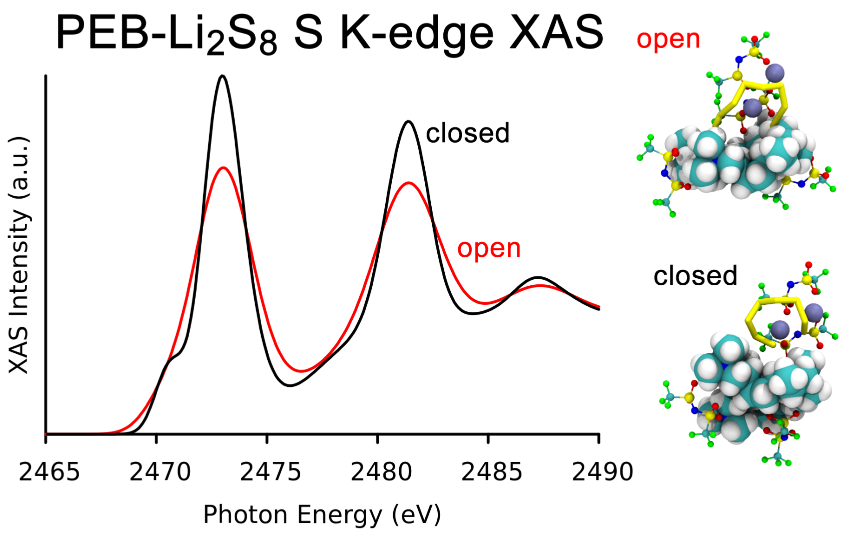

Supplementary Figure 6 | Simulated sulfur K-edge X-ray absorption near edge structure spectra of PEB-1/Li_2S_8 with closed and open polysulfide complexes, with respect to Li^+ coordination. These calculations support the assessment of features in the experimentally observed S K-edge XAS as arising from a ring-opened polysulfide configuration.

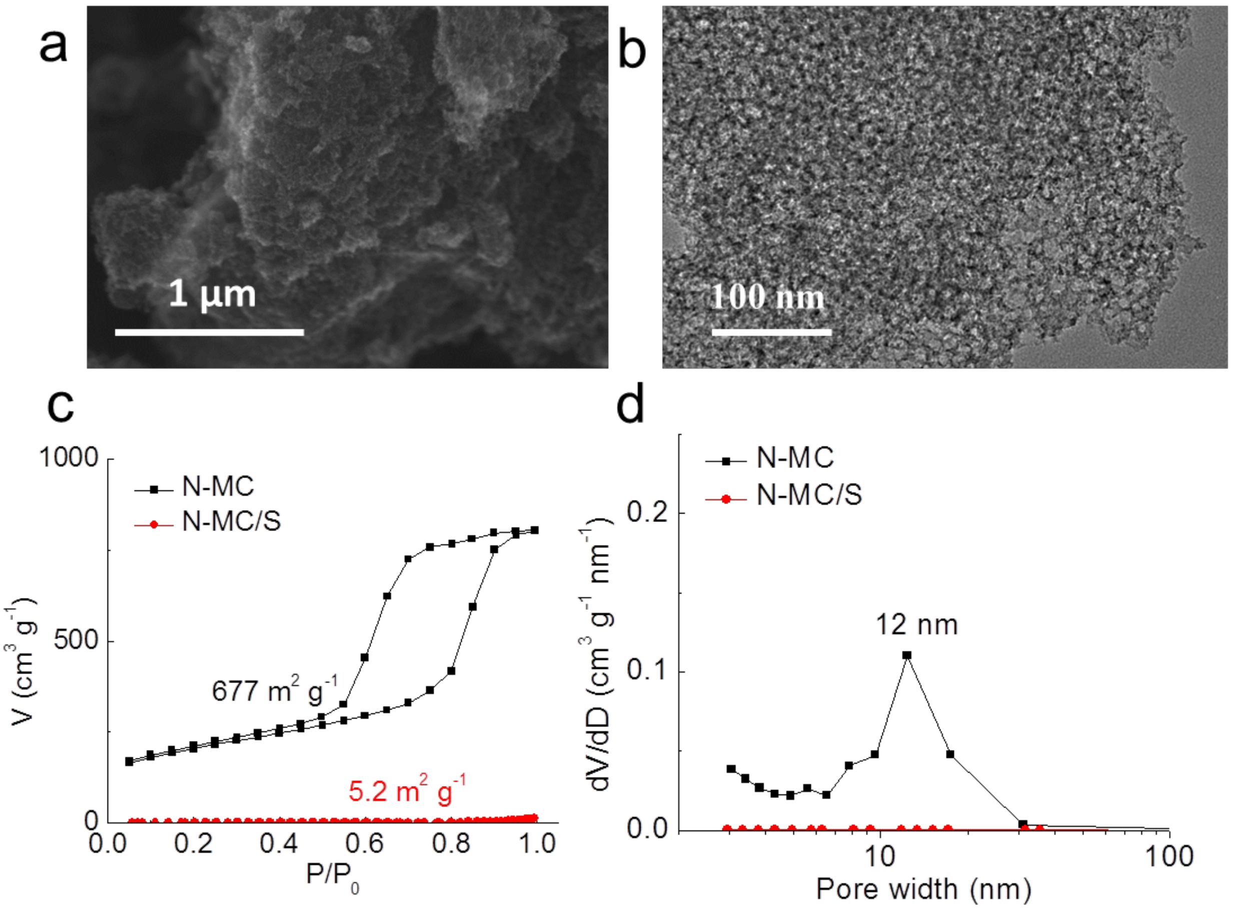

Supplementary Figure 7 | Characterization of N-Doped Mesoporous Carbon (N-MC) and N-MC/S. **a**, SEM of N-MC. **b**, TEM of N-MC. **c**, Nitrogen adsorption isotherms of N-MC and N-MC/S. **d**, BJH pore size distribution of N-MC and N-MC/S.

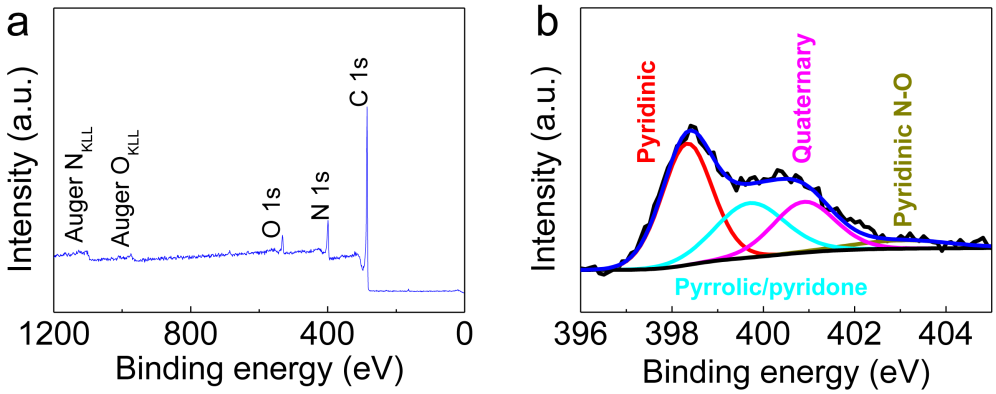

Supplementary Figure 8 | XPS analysis of N-MC hosts for sulfur active materials. a, Survey scan noting the presence of N, C, and O elements in the material. **b**, Analysis of the N 1s signal, noting the presence of un-oxidized and oxidized sp^2 motifs, and quaternized sp^3 motifs.

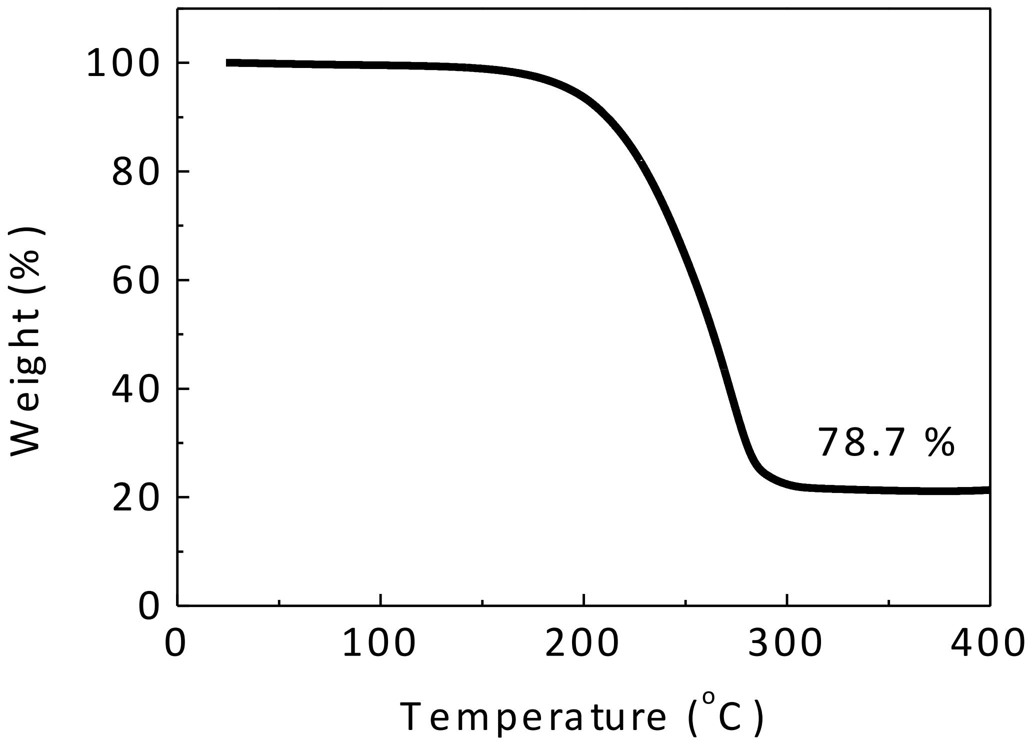

Supplementary Figure 9 | TGA analysis of N-MC/S composite.

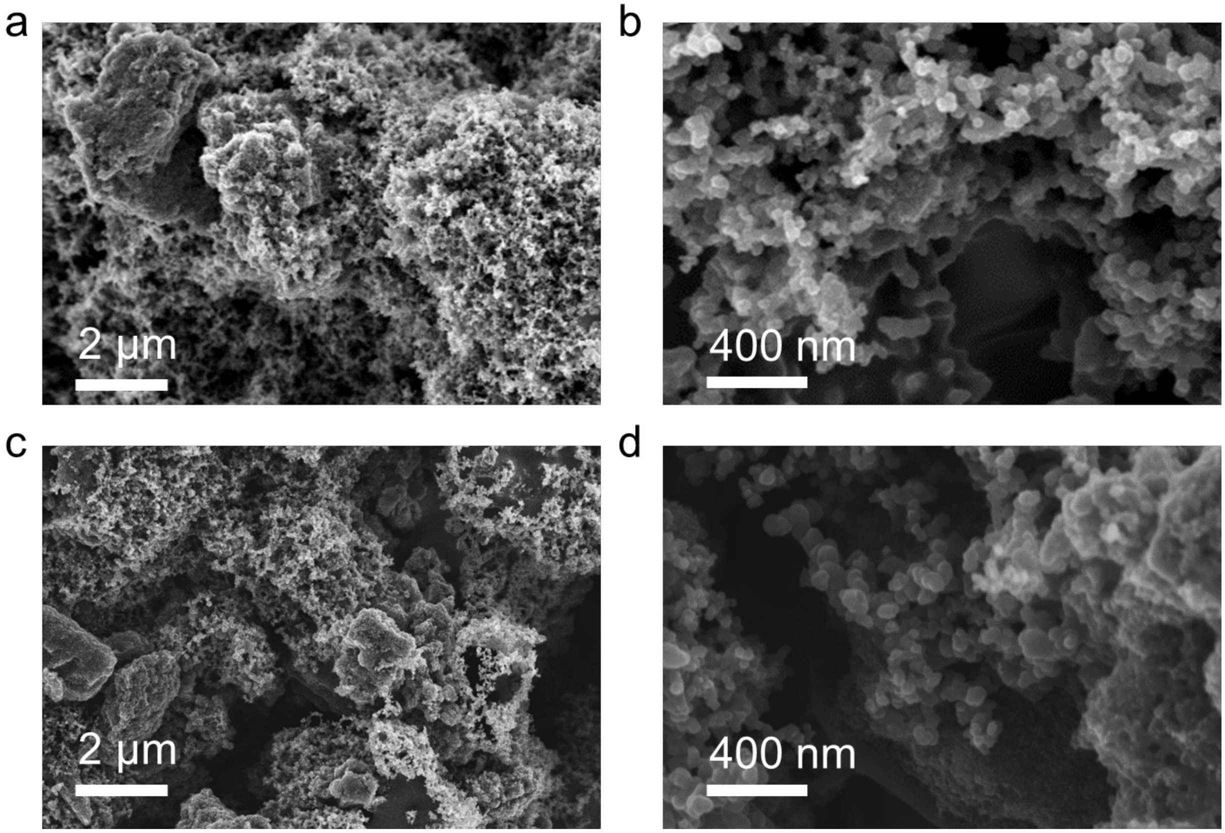

Supplementary Figure 10 | SEM images of sulfur cathodes with different binders. a-b, PEB-1**. c-d,** PVDF**.**

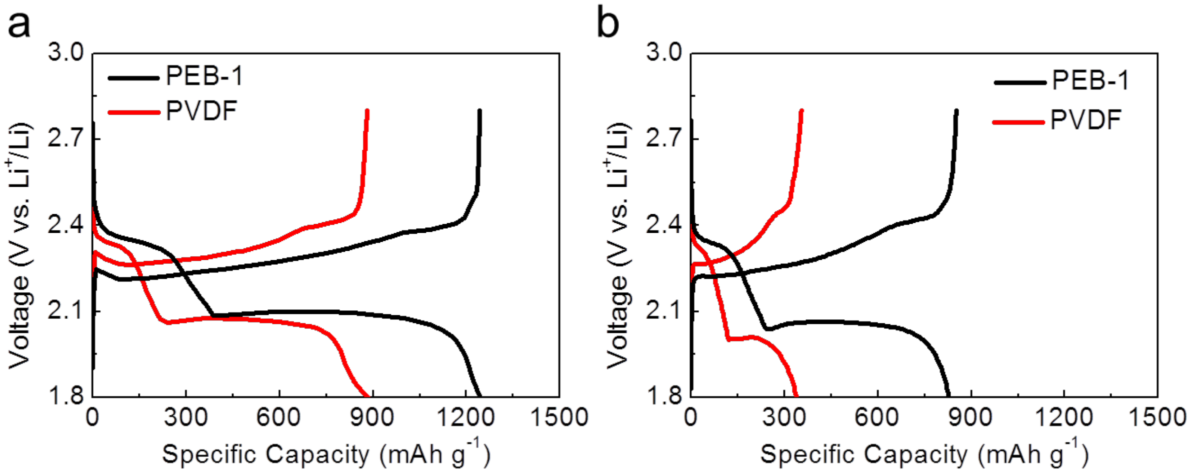

Supplementary Figure 11 | Discharge-Charge voltage profiles of PVDF and PEB-1 cells with a sulfur loading of 1.2 mg cm^{-2} deposited onto an aluminum current collector. a, 1st and **b**, 200th cycles.

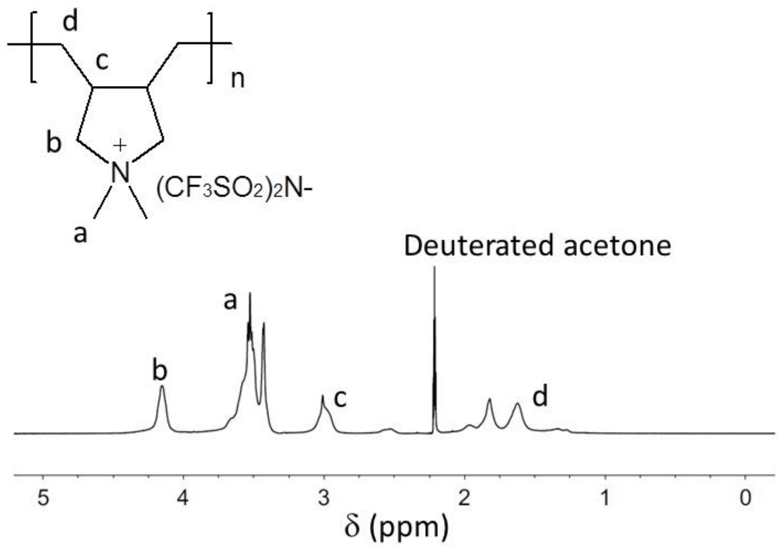

Supplementary Figure 12 | 500 MHz ^{1}H NMR spectra of PEB-1.

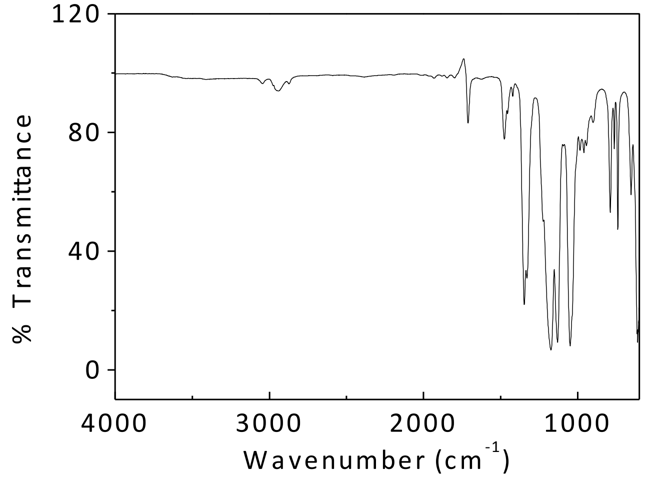

Supplementary Figure 13 | FT-IR spectrum of PEB-1.

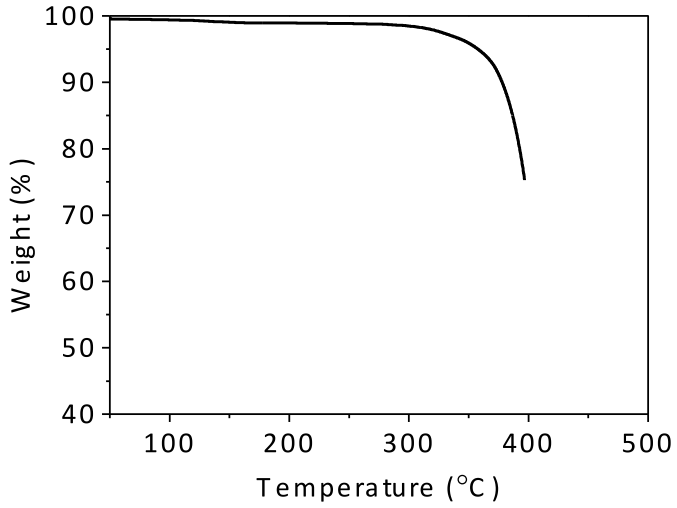

Supplementary Figure 14 | TGA analysis of PEB-1.

Supplementary Table 1 | Comparison of recently reported binders for Li–S batteries.

Sample	Sulfur Loading (mg cm^{-2})	Areal Capacity (mA h cm^{-2})	Rate	Discharge Capacity (mA h g^{-1} sulfur)	Cycle Number	Current Collector	Reference
Guar Gum+Xanthan Gum	19.8	26.4	C/40	1333	5	Ni Foam	32
Carbonyl-β-Cyclodextrin	3	4.5	C/5	1500	50	Carbon-Coated Al Foil	33
Nafion/PVP	1.7	2.30	C/10	1350	200	Al Foil	34
LA132	1	0.9	C/5	900	50	Al Foil	35
Gelatin	2.0	2.3	0.12 C	1150	50	Al Foil	36
Guar Gum	0.7	0.74	C/5	1050	150	Al Foil	37
AFG	1.2	0.75	1C	625	200	Ni Foam	38
PAMAC	3	3.15	C/25	1049	105	Carbon-Coated Al Foil	39
PEO	1.5	1.88	C/10	1256	100	Al Foil	40
PFM	0.3	0.36	C/10	1200	150	Al Foil	41
PVP	2	1.6	C/5	800	500	Carbon Fiber Paper	42
**PEB-1**	**8.1**	**8.13**	**C/5**	**1004**	**100**	**Carbon Fiber Paper**	**This Work**

Supplementary Table 2 | Elemental analysis of PEB-1.

	% Estimated	% Found
C	29.56	29.43
H	3.97	3.87
N	6.89	6.82
O	15.78	16.15

Supplementary References

1. Mayo, S. L., Olafson, B. D. & Goddard, W. A. Dreiding: A generic force field for molecular simulations. *J. Phys. Chem.* **94,** 8897–8909 (1990).

2. Pascal, T. A.*, et al.* Liquid sulfur impregnation of microporous carbon accelerated by nanoscale interfacial effects. *Nano Lett.* **17,** 2517–2523 (2017).

3. Chen, J. & Martínez, T. J. QTPIE: Charge transfer with polarization current equalization. A fluctuating charge model with correct asymptotics. *Chem. Phys. Lett.* **438,** 315–320 (2007).

4. Rappe, A. K. & Goddard, W. A. Charge equilibration for molecular dynamics simulations. *J. Phys. Chem.* **95,** 3358–3363 (1991).

5. Pascal, T. A.*, et al.* X-ray absorption spectra of dissolved polysulfides in lithium–sulfur batteries from first-principles. *J. Phys. Chem. Lett.* **5,** 1547–1551 (2014).

6. Pascal, T. & Prendergast, D. Mf3 - a fluctuating charge forcefield for simulating energy relevant materials. *In preparation***,** (2017).

7. Plimpton, S. Fast parallel algorithms for short-range molecular-dynamics. *J. Comput. Phys.* **117,** 1–19 (1995).

8. Maiti, P. K. P., Tod A.; Vaidehi, Nagarajan; Goddard, William A. Understanding DNA based nanostructures. *J. Nanosci. Nanotechnol.* **7,** 1712–1720 (2007).

9. Maiti, P. K., Pascal, T. A., Vaidehi, N., Heo, J. & Goddard, W. A. Atomic-level simulations of Seeman DNA nanostructures: The paranemic crossover in salt solution. *Biophys. J.* **90,** 1463–1479 (2006).

10. Maiti, P. K., Pascal, T. A., Vaidehi, N. & Goddard, I. I. I. W. A. The stability of seeman jx DNA topoisomers of paranemic crossover (px) molecules as a function of crossover number. *Nucleic Acids Res.* **32,** 6047–6056 (2004).

11. Shinoda, W., Shiga, M. & Mikami, M. Rapid estimation of elastic constants by molecular dynamics simulation under constant stress. *Phys. Rev. B* **69,** 134103 (2004).

12. Martyna, G. J., Tobias, D. J. & Klein, M. L. Constant-pressure molecular-dynamics algorithms. *J. Chem. Phys.* **101,** 4177–4189 (1994).

13. Parrinello, M. & Rahman, A. Polymorphic transitions in single crystals: A new molecular dynamics method. *J. Appl. Phys.* **52,** 7182–7190 (1981).

14. Mark, E. T., José, A., Roberto, L.-R., Andrea, L. J. & Glenn, J. M. A Liouville-operator derived measure-preserving integrator for molecular dynamics simulations in the isothermal–isobaric ensemble. *J. Phys. A: Math. Gen.* **39,** 5629 (2006).

15. Barducci, A., Bussi, G. & Parrinello, M. Well-tempered metadynamics: A smoothly converging and tunable free-energy method. *Phys. Rev. Lett.* **100,** 020603 (2008).

16. Bussi, G., Laio, A. & Parrinello, M. Equilibrium free energies from nonequilibrium metadynamics. *Phys. Rev. Lett.* **96,** 090601 (2006).

17. Pascal, T. A.*, et al.* Finite temperature effects on the X-ray absorption spectra of lithium compounds: First-principles interpretation of X-ray raman measurements. *J. Chem. Phys.* **140,** 034107–034121 (2014).

18. Prendergast, D. & Galli, G. X-ray absorption spectra of water from first principles calculations. *Phys. Rev. Lett.* **96,** 215502 (2006).

19. England, A. H., Duffin, A. M., Schwartz, C. P., Uejio, J. S., Prendergast, D. & Saykally, R. J. On the hydration and hydrolysis of carbon dioxide. *Chem. Phys. Lett.* **514,** 187–195 (2011).

20. Vanderbilt, D. Soft self-consistent pseudopotentials in a generalized eigenvalue formalism. *Phys. Rev. B* **41,** 7892–7895 (1990).

21. Giannozzi, P.*, et al.* Quantum espresso: A modular and open-source software project for quantum simulations of materials. *J. Phys.: Condens. Matter* **21,** 395502 (2009).

22. Shirley, E. L. Optimal basis sets for detailed brillouin-zone integrations. *Phys. Rev. B* **54,** 16464 (1996).

23. Prendergast, D. & Louie, S. G. Bloch-state-based interpolation: An efficient generalization of the shirley approach to interpolating electronic structure. *Phys. Rev. B* **80,** 235126 (2009).

24. Taillefumier, M., Cabaret, D., Flank, A.-M. & Mauri, F. X-ray absorption near-edge structure calculations with the pseudopotentials: Application to the K-edge in diamond and α-quartz. *Phys. Rev. B* **66,** 195107 (2002).

25. Jiang, P.*, et al.* Experimental and theoretical investigation of the electronic structure of Cu_2O and CuO thin films on Cu(110) using X-ray photoelectron and absorption spectroscopy. *J. Chem. Phys.* **138,** 024704–024706 (2013).

26. Rühl, E., Flesch, R., Tappe, W., Novikov, D. & Kosugi, N. Sulfur 1s excitation of S_2 and S_8: Core–valence– and valence–valence–exchange interaction and geometry-specific transitions. *J. Chem. Phys.* **116,** 3316–3322 (2002).

27. Cohen, A. J., Mori-Sanchez, P. & Yang, W. T. Fractional charge perspective on the band gap in density-functional theory. *Phys. Rev. B* **77,** 115123 (2008).

28. Mori-Sanchez, P., Cohen, A. J. & Yang, W. T. Localization and delocalization errors in density functional theory and implications for band-gap prediction. *Phys. Rev. Lett.* **100,** 146401 (2008).

29. Fuchs, F., Furthmüller, J., Bechstedt, F., Shishkin, M. & Kresse, G. Quasiparticle band structure based on a generalized Kohn-Sham scheme. *Phys. Rev. B* **76,** 115109 (2007).

30. Aulbur, W. G., Jönsson, L. & Wilkins, J. W. Quasiparticle calculations in solids. *Solid State Physics* **54,** 1–218 (1999).

31. Pascal, T. A., Pemmaraju, C. D. & Prendergast, D. X-ray spectroscopy as a probe for lithium polysulfide radicals. *Phys. Chem. Chem. Phys.* **17,** 7743–7753 (2015).

32. Liu, J.*, et al.* Exploiting a robust biopolymer network binder for an ultrahigh-areal-capacity Li-S battery. *Energy Environ. Sci.* **10,** 750–755 (2017).

33. Wang, J., Yao, Z., Monroe, C. W., Yang, J. & Nuli, Y. Carbonyl-β-cyclodextrin as a novel binder for sulfur composite cathodes in rechargeable lithium batteries. *Adv. Funct. Mater.* **23,** 1194–1201 (2013).

34. Wang, Q.*, et al.* Layer-by-layer assembled C/S cathode with trace binder for Li–S battery application. *ACS Appl. Mat. Interfaces* **7,** 25002–25006 (2015).

35. Hong, X.*, et al.* On the dispersion of lithium-sulfur battery cathode materials effected by electrostatic and stereo-chemical factors of binders. *J. Power Sources* **324,** 455–461 (2016).

36. Sun, J., Huang, Y., Wang, W., Yu, Z., Wang, A. & Yuan, K. Application of gelatin as a binder for the sulfur cathode in lithium–sulfur batteries. *Electrochim. Acta* **53,** 7084–7088 (2008).

37. Lu, Y.-Q.*, et al.* Achieving high capacity retention in lithium–sulfur batteries with an aqueous binder. *Electrochem. Commun.* **72,** 79–82 (2016).

38. Chen, W.*, et al.* A new type of multifunctional polar binder: Toward practical application of high energy lithium sulfur batteries. *Adv. Mater.* **29**, 1605160 (2017).

39. Zhang, S. S. Binder based on polyelectrolyte for high capacity density lithium/sulfur battery. *J. Electrochem. Soc.* **159,** A1226–A1229 (2012).

40. Cheon, S.-E.*, et al.* Structural factors of sulfur cathodes with poly(ethylene oxide) binder for performance of rechargeable lithium sulfur batteries. *J. Electrochem. Soc.* **149,** A1437–A1441 (2002).

41. Ai, G.*, et al.* Investigation of surface effects through the application of the functional binders in lithium sulfur batteries. *Nano Energy* **16,** 28–37 (2015).

42. Seh, Z. W., Zhang, Q., Li, W., Zheng, G., Yao, H. & Cui, Y. Stable cycling of lithium sulfide cathodes through strong affinity with a bifunctional binder. *Chem. Sci.* **4,** 3673–3677 (2013).
